# A Game-Theory Based Incentive Framework for an Intelligent Traffic System as Part of a Smart City Initiative

**DOI:** 10.3390/s17122874

**Published:** 2017-12-11

**Authors:** Haibo Mei, Stefan Poslad, Shuang Du

**Affiliations:** 1 School of Aeronautics and Astronautics, University of Electronic Science and Technology of China, Chengdu 610051, China; sdu@uestc.edu.cn; 2 School of Electronic Engineering and Computer Science, Queen Mary University of London, London E1 4NS, UK; stefan.poslad@qmul.ac.uk

**Keywords:** intelligent transportation system, incentive, evolutionary game theory, decision tree

## Abstract

Intelligent Transportation Systems (ITSs) can be applied to inform and incentivize travellers to help them make cognizant choices concerning their trip routes and transport modality use for their daily travel whilst achieving more sustainable societal and transport authority goals. However, in practice, it is challenging for an ITS to enable incentive generation that is context-driven and personalized, whilst supporting multi-dimensional travel goals. This is because an ITS has to address the situation where different travellers have different travel preferences and constraints for route and modality, in the face of dynamically-varying traffic conditions. Furthermore, personalized incentive generation also needs to dynamically achieve different travel goals from multiple travellers, in the face of their conducts being a mix of both competitive and cooperative behaviours. To address this challenge, a Rule-based Incentive Framework (RIF) is proposed in this paper that utilizes both decision tree and evolutionary game theory to process travel information and intelligently generate personalized incentives for travellers. The travel information processed includes travellers’ mobile patterns, travellers’ modality preferences and route traffic volume information. A series of MATLAB simulations of RIF was undertaken to validate RIF to show that it is potentially an effective way to incentivize travellers to change travel routes and modalities as an essential smart city service.

## 1. Introduction

If we are to achieve more beneficial and cohesive personal, business and societal goals, in the face of increasing global challenges such as an increasing sedentary lifestyle for citizens, the promotion of physical environment sustainability, a reduction in poverty, better physical and social living conditions, etc., we need to change our behaviour and consider how to do this more effectively. One plausible approach to achieve this is to consider incentivizing behaviour shifts. An incentive is something that motivates us as society members to select and perform specific actions, usually to achieve beneficial personal, business and societal goals. As our application and focus are on the use of transport as an action, we consider how incentives can beneficially improve the use of transport.

A lack of exercise is a major cause of heart disease [1]; hence, a good example of a personal transport incentive is to choose how to use human-powered transport between home and work more effectively. For example, in a study in Australia, men who cycled to work were found to be significantly less likely to be overweight and obese compared with those driving to work [2]. A common use of incentives in business is to facilitate economic gain by an individual or via competition or cooperation within an organization. To help improve the attainment of organization goals, such as increased productivity, prizes of pre-paid travel for MICE (meetings, incentives, conferences and exhibitions) in an exotic location can be offered [3]. A beneficial transport incentive for society is to promote the use of off-peak travel and hence to lower the peak air quality and high-carbon emissions pertaining to peak-time commuting [4]. Whilst many aspects of transport use are predictive or deterministic in nature in terms of being circadian or daily, weekly and seasonally driven, some transport use characteristics may be more dynamic and less temporally deterministic, e.g., road traffic congestion can be more effectively modelled if we handle not just the deterministic temporal aspect, but also the non-deterministic temporal aspects [5]. These time-driven aspects begin already to illustrate the need for a multi-dimensional, context-driven, individualized or personalized, design for travel incentives. We can also consider that a higher daily travel cost for peak-time public transport coupled with over-crowding versus a lower daily cost for off-peak travel with less crowding can be perceived as both a penalizing incentive for using peak travel versus a reward incentive for using off-peak travel.

Hence, one of the challenges when designing incentives for off-peak versus peak travel is how to effectively leverage the use of the temporal context when organizing travel. There are several additional dimensions that need to be considered when designing incentives. Different travellers have different (personal) preference restrictions based on their location context that affect their dominant transport mode use, e.g., numerous studies have demonstrated that living in higher density, mixed transport use neighbourhoods is associated with less car use compared to living in low density, suburban environments [6]. Hence, offering a global, or the same incentive, to each participant, e.g., to maximize the use of public transport to reach an end destination, by the shortest time and at an affordable cost appears disadvantageous for rural travellers. Often, the goal to reach an end destination is to travel by the shortest time at an affordable cost. If we consider the effectiveness of personalized travel incentives in isolation, and to be static, new secondary-order effects can arise, i.e., the increased demand for the use of inexpensive public travel routes with respect to time and location may lead to additional overcrowding at specific times and locations because the service supply resources cannot meet the demand [7]. Thus, we need to also consider the cooperative and competitive behaviours of travellers as a collective when proposing travel incentives, else new, second-order less desirable norms could arise such as overcrowding on certain peak travel routes that may violate some travellers’ personal preferences to use these even though societal sustainable travel goals are achieved. The authors in [3] have noted that for the area of incentivized travel, it is particularly lacking in academic research and remains without a strong theoretical foundation. One promising approach to achieve a balance between multiple goal dimensions taking into account group participants’ behaviour is to model the use of transport using game theory and machine learning methods.

Numerous Intelligent Transport Systems (ITS) have been researched and developed that support that personalized travel plans is advantageous. For example, common personalization preferences are to choose the start and end destination, the transport mode and for route and destination information to be adapted and to be presented in this way according to these preferences [8,9]. However, it is challenging to design incentives to shift travellers’ behaviours, for example to use more sustainable transport, away from their preconceived or default transport usage. For example, if by default we offer the same universal or global incentive to shift their use of their habitual travel plan to use more sustainable transport modes, some travellers may feel less inclined to do so because of their temporal, local or personal constraints and preferences. This is a complex problem. We also often tend to have more than one personal transport use goal [10], so we need to consider how we need to more effectively design multi-dimensional incentives, e.g., ones that use multiple modes and that are context driven, as well as personalized ones with respect to travellers’ preferences and location and the real-time travel situation.

There exists numbers of ITSs designed as part of the smart city initiative to incentivize travellers to use efficient and eco-friendly transportation [11]. However, when generating incentives for travellers, few ITSs take individual travellers’ preferences and incentive multi-dimensional goals into consideration. Some ITSs simply broadcast traffic signals, state information or non-personalized travel guidance to travellers [12]. Some systems, such as the Advanced Traveller Information Systems [13] and Cooperative Multi-agent Transportation Management and Route Guidance System [14], were proposed to meet the incentive challenge. However, in [13,14], the incentives are mostly based on traffic state information, and the authors did not fully take travellers’ preferences combined with traffic state information into consideration to achieve multi-dimensional travel goals. To the best of our knowledge, little or no work has been undertaken within ITS to generate personalized, situation-aware travel incentives that also model and account for social or group behaviour.

The use of machine learning and game theory has been proposed as a promising candidate to solve the computing intelligence and multi-dimensional goal problem [15,16]. Following this trend, we designed a Rule-based Incentive Framework (RIF) to intelligently generate context-driven incentives for travellers, while considering the multi-dimensional goals. RIF supports personal incentives through the use of the Iterative Dichotomiser 3 (ID3) algorithm [15] as its classical machine learning-based decision tree algorithm and evolutionary game theory [17] as its game theory method. Through the generation of personalized incentives, RIF can intelligently and efficiently re-allocate traffic route capacity over time and space while adhering to any individual traveller’s preferences for transport modality and route, according to multi-dimensional incentive goals. RIF utilizes personal mobility patterns [4], traffic volume information and traveller’s modality preferences as three of the main sources of real-time inputs for the utility calculations and the decision tree-related processing. In summary, the primary contributions of this paper are to:

1. Propose a rule-based incentive framework to solve the personalized context-driven incentive problem, whilst achieving personalized travel preference, travel efficiency and eco-friendly global transportation as the multi-dimensional goals.
2. Apply the decision tree as a machine learning algorithm to help generate personal incentives for travellers in RIF, in part based on the historical travel information.
3. Apply Population Evolution (PE) theory as a classic evolutionary game theory to address the personal incentive problem in RIF. By PE, the best fit personal incentives are generated for travellers based on the output of the Analytic Hierarchy Process (AHP) and the comparisons of the incentive utilities.
4. Provide a theoretical and numerical analysis and comparisons of the decision tree and population evolution-based incentive solutions in RIF.

The remainder of this paper is organized as follows. In Section 2, we give the background and related work. In Section 3, we describe the problem formulation. In Section 4 and Section 5, we present the RIF based on the use of the decision tree and PE to solve the personal incentive problem. In Section 6, we discuss and analyse the pros and cons of using the decision tree and PE methods. Our simulation results are presented in Section 7. In Section 8, we present our conclusions and future work.

## 2. Background and Related Work

### 2.1. The Context-Driven and Multi-Dimensional Incentives for ITS

In practice, an efficient travel incentive is context-driven, situation-aware and personalized. In this paper, we specify incentives that propose travel route and modality changes to travellers at specific time points. Travel route and modality can be chosen by a traveller based on a traveller’s personal preferences and route real-time volume information. For example, a traveller’s preference for a travel route may be a combination of the shortest, fastest and picturesqueness route [18]. Meanwhile, a traveller’s preference for travel modality could be car or bus, etc. Furthermore, personal preferences may change on-route and need to be dynamic. Therefore, it is a complex challenge to generate effective incentives for travellers in an ITS.

From the travellers’ perspective, an incentive can be classified as a reward incentive or a penalty incentive [4]. A reward incentive intends to change traveller’s traffic behaviours through a positive stimulation either physically or psychologically. For example, SUNSET: a sustainable social networking sevices for transport in Europe [4], INSINC: a platform for managing peak demand in public transit in Singapore [19] and INSTANT: an incentive mechanism for decongesting the roads in Bangalore [20] all employ reward incentives. All of these projects offer travellers rewards (reward points, cash, gifts or traffic ticket discounts) when they finish travel challenges or goals, such as taking trains pre-peak time and changing the route to avoid congestion. For penalty incentives, traffic managers can issue congestion tax, fuel tax and use tolls to reduce traffic congestion and to achieve a designated modality bias. In reality, penalty incentives appear relatively easy to realize and are widely used, e.g., to use higher peak time versus far lower off-peak travel fares, whilst the use of reward incentives seems to date to be somewhat still in its infancy [21].

With respect to their issue conditions, incentives can be classified into proactive incentives and reactive ones [4]. A proactive incentive is designed to influence travellers’ behaviours before their traffic activity happens. In contrast, a reactive incentive is designed to influence travellers’ behaviours during their journey. To aid travellers to satisfy their individual goals and to meet global societal goals such as reducing traffic congestion, we focus on the design of incentives to be proactive and reward based. An incentive is linked to transport use or selection, e.g., to the use of a specific route and transport mode at a specific time point. To promote a reward-type incentive, projects such as SUNSET, INSINC and INSTANT grant points or gifts to travellers, if they accept the incentives. Similarly, the authors in [22] considered the incentives to be offered to location-tracked private car users to change their departure time during daily commute in order to reduce congestion and their travel time. In [23], they considered personalized incentives provision in a multimodal transport system based on the travellers with eight attitudinal profiles to change their route to a less congested one, where users’ smartphones act as mobile sensors and message distributors. In [24], they looked at what monetary incentives would cause commuters to change their daily commute in San Francisco, CA, USA. The authors in [25] considered the application of gamification to get more users to switch to use more sustainable travel options. None of this work considers how one person’s travel strategy is dependent on the strategic travel choices of others, i.e., the application of game theory. Without this, new, less optimal overall system states can more easily arise, such as congestion on previously un-congested routes.

### 2.2. Decision Tree

Decision trees have been comprehensively studied in [26] and further applied to solve a variety of ITS problems [27,28,29]. In theory, a decision tree can effectively help RIF to support personalized incentives, i.e., to find a best fit discrete function for a Decision Support System (DSS). The discrete function can be expressed in the form of a tree using a conjunction and disjunction mixed formula. Using a discrete function, a specific decision making problem can be solved according to its input parameters. To the best of our knowledge, there is no current work that investigates the application of a decision tree to model and solve the personalized travel problem. In this paper, we apply the decision tree as part of RIF to model personalized travel incentives according to travellers’ historical travel information.

In practice, many currently-used decision tree algorithms tend to be variants of the ID3 algorithm, which is a classic algorithm, as proposed in [26]. By learning travellers’ historical sample data, ID3 constructs a top-down decision tree, i.e., a discrete function, by evaluating which criterion is the best one to be put at a specific node in the decision tree to work as the decisional criterion. In Section 4, the ID3 algorithm will be applied to help solve the personal incentive problem.

### 2.3. Game Theory Used for ITS

Although a machine learning algorithm such as a decision tree can help solve the personalized incentive problem, it heavily depends on the amount of historical travel data and uses complex computation processes, which may cause performance bottlenecks. Fortunately, game theory provides a better alternative solution. In practice, although game theory and gamification may be used interchangeably, these are not the same thing. Game theory analyses strategic situations where one person’s strategy is dependent on the strategic choices of others, e.g., the classic Prisoner’s dilemma. In contrast, gamification uses game play mechanics to get people more involved in an activity and for example uses rewards to encourage particular behaviours. Our focus is on the application of game theory rather than gamification applied to ITS. Game theory provides a powerful means for an ITS, by which it can act on behalf of travellers and perform a negotiation between travellers. Specifically, by game theory, a traveller or his/her travel agency can learn and manage his/her optimum strategy by observing the actions of other travellers, leading to an equilibrium state where most of the travellers receive a maximum possible payoff. Game theory has been comprehensively studied in [16,30]. Based on the survey, games can be classified into different types such as: non-cooperative and cooperative games. In [31,32,33], classic game theories, including Nash equilibrium and Stackelberg equilibrium, have been applied to transportation management, normally in a non-cooperative approach, which focuses on the benefits of individual travellers or traffic agents. However, because all travellers as game players are selfish, it is difficult for classic games to find a systematic transportation optimization that can benefit not only a single traveller, but also the global transportation goals, such as reducing congestion in a region, maximizing public transportation use and energy efficiency, etc. In [34], cooperative game theories have been discussed including stochastic games (Q-learning) and evolutionary games in order to avoid a non-optimal traffic equilibrium. Compared to classic games, evolutionary game theory [35] applies principles in evolving populations of life forms in biology to fields such as the economy, network engineering and social sciences. Evolutionary game theory can easily find an optimization that guarantees each player’s payoffs to reach a global optimal equilibrium. Thus, evolutionary game theory potentially is more able to help ITSs to realize sustainable transportation in smart cities. Conversely, little work has so far applied evolutionary game theory to enable incentive-based ITSs.

In this paper, we propose to employ a well-known evolutionary game theory, more specifically Population Evolution (PE) [17], coupled with an Analytic Hierarchy Process (AHP) [36] method to help make real-time incentive decisions in RIF. With PE, the personal incentives can be generated for travellers based on the outputs of AHP and the comparison of real-time incentive utilities (i.e., payoffs). In theory, a PE-based incentive solution could give a better performance compared to a decision tree. This will be discussed in the analysis part of this paper.

### 2.4. Population Evolution

PE is a well-known evolutionary game theory, which has been applied in various fields. For example in [17,37,38], the authors utilized PE to solve a network selection and an adaptive routing issue. PE basically simulates the population growth of species, such as travellers of a route, in a physical region. It follows in principle that if there are more resources (a higher utility), the population will grow (as more individuals join). Equation (1) is used to formulate this principle.

(1) $$\begin{array}{c} {\dot{x}}_{k}^{g}=\sigma \times x_{k}^{g}\times \left(U_{k}^{g}-{\bar{U}}_{g}\right) \end{array}$$

Equation (1) indicates that for each period, the individuals observe the overall utility of choosing strategy *k*, $U_{k}^{g}$ in group *g*, and the average utility of the entire population, ${\bar{U}}_{g}$ in group *g*. In the next step, $x_{k}^{g}$ as the utility of an individual choosing strategy *k* is adapted for ${\dot{x}}_{k}^{g}$ accordingly. The higher the utility of strategy *k*, the more individuals will choose it in the group. A strategy *k* could be for leaving this group versus staying in the group or for joining the group. The utility adaptation is carried out with the help of replicator dynamics where σ is the gain for the rate of strategy adaptation. It is obvious that PE is normally used for decision making based on utility comparisons.

PE is applicable to the personal incentive problem of this paper. This is because we can take an incentive with related traffic route and modality as a block of traffic resources. For an incentive, its related traffic route can handle a number of travellers’ use of transportation modalities. If the route is less congested for travellers using one kind of modality, it supports an incentive with a high utility to persuade more travellers to join this route using that modality. This incentive principle can be modelled by Equation (1). In Section 5, we will apply PE to solve the personal incentive problem coupled with the use of the AHP method.

## 3. Problem Formulation

### 3.1. User Scenario and Incentive

**Figure 1:**
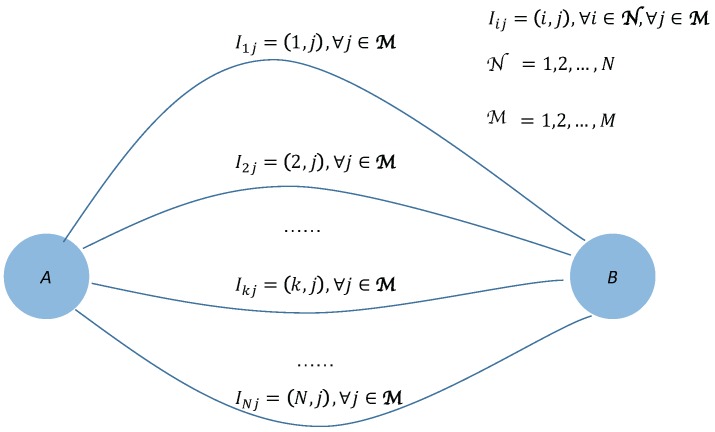
Rule-based Incentive Framework (RIF) model: travellers competing to select incentives between two traffic points.

In this paper, we introduce a typical user scenario for RIF to work with, which is shown in Figure 1. Then, the problem formulation is presented based on this scenario. In Figure 1, there are two traffic points *A* and *B* connected by *N* candidate routes grouped as N=1,2,…,N. Travellers at one time can travel in one of the routes from group N using one of the *M* possible modalities, e.g., foot, cycle, car, bus, etc., from group M=1,2,…,M. For a travel route *i*, its traffic volume is composed of its incoming travellers and existing traffic $V_{i}$, and the maximal capacity of the route *i* is denoted as $V_{i}^{max}$. In practice, through RIF, a traveller *t* with specific travel route and modality preferences could compete for the available capacity resources within the candidate routes from N while using a specific modality from M between traffic points *A* and *B*.

According to the user scenario shown in Figure 1, RIF should work as an intelligent traffic instructor for the travellers between traffic points *A* and *B*. In this paper, RIF shapes a series of incentives issued to travellers to suggest to them a less congested route and a better modality to save travel time, cost and pollution. In order to do so, RIF has to consider travellers’ preferences and real-time traffic situations, which will be summarized as incentive criteria later. Two important incentive design issues are first when and under what conditions will RIF shape and issue incentives to travellers and, second, what real-time traffic information is available to travellers. As discussed before, the incentive considered in this paper is of the proactive type. Therefore, RIF will shape incentives to those travellers when they start travelling from traffic point *A* to *B* (or from *B* to *A*) at a designated time point. Of course, there are other cases where incentives could be shaped and issued to travellers either proactively or reactively in a temporary or un-predictable manner, like a route change becomes more preferable than sticking with a problematic route. In order to illustrate the use of RIF, we consider the case given in Figure 1.

Accordingly, we use $I_{ij}=\left(i,j\right),\forall i\in N,\forall j\in M$ to denote the incentives to be issued to travellers in RIF. An incentive $I_{ij}$ suggests its recipient traveller to travel through route *i* by modality *j* between traffic points *A* and *B* at a specific time point. Obviously, for a specific route *k*, its involved incentives could be $I_{kj}=\left(k,j\right),\forall j\in M$. The objective of RIF therefore is to guide any traveller *t*, grouped as T=1,2,…,T, to find his/her best fit incentive $I_{ij}$ between traffic points *A* and *B* following certain incentive criteria at a time. We use $\alpha_{tij}$ to denote whether an incentive $I_{ij}$ is issued to traveller *t* ($\alpha_{tij}=1$) or not ($\alpha_{tij}=0$) in RIF. Therefore, $\alpha ={\left[\alpha_{tij}\right]}_{T\cdot N\cdot M}$ is the output that RIF tries to reach. In this paper, we think about the personal incentive issuing at a specific, i.e., static time point, therefore, in rest part of this paper, we will not mention time any more.

### 3.2. Incentive Criteria and Weighting

When RIF generates incentives to travellers, the incentive criteria under consideration in this paper include:

1. A traveller’s preference for a route that indicates the traveller’s willingness to take up a suggestion for an alternative route.
2. A traveller’s preference for a modality that indicates the traveller’s willingness to take up a suggestion for an alternative modality.
3. Current route traffic volume that determines whether a traveller is in congested traffic or not.

Such incentive criteria, i.e., travel route, modality and traffic volume, were selected with the aim to balance between the need for guiding traffic travellers to select other travel options (route and modality) in the event of congestion while maintaining the overall traveller satisfaction. If a traveller has a high willingness to change travel route or modality, a suggestion to change the route will not adversely affect the traveller’s satisfaction. On the other hand, suggesting a change to less willing travellers will lower their satisfaction. In reality, travellers can be provided with a smartphone application or related device that tracks their personal mobility and travel patterns. For instance, the system can gain an insight into one traveller’s personal mobility profile by referring to the traveller’s frequent locations, habitual routes and modalities. In addition, the system can acquire personal preferences from a traveller, such as one’s modality and route preferences and his/her tolerance towards congestion, using an appropriate Human-Computer Interaction (HCI) design, such as a questionnaire. In practice, measurements of traffic volume can be taken in real time using appropriate sensors such as inductive loop detectors under the road surface at traffic lights. For traffic that exhibits daily, seasonal or other patterns, historical data can be analysed to provide short-term traffic predictions.

To solve the personal incentive problem, we can weight each criteria to represent the importance of a criterion to a traveller, which is according to the sensitivity of the traveller to the criterion using AHP. AHP is a methodology that has been widely used for complex decision support in different fields such as government, business, industry, healthcare and education [36]. In this paper, the incentive-related decision making can be a very complex process, particularly when a number of dependent elements, i.e., criteria, including travel route, modality and route volume, are involved. These elements are dependent because they are co-related highly in transportation model. For example, there might not be a public transport route between the origin and destination, so no bus modality is associated with a specific route. Same goes for volume and modality, etc. In practice, it is complicated and difficult to make correct incentive decisions considering the dependent elements, even when these elements in ITS are tangible, carefully estimated, and well understood for the personal incentive problem at hand. As each element has a different influence on the final incentive decision, there is no absolute or correct personal incentive decision. Fortunately, AHP can help the personal incentive decision making to find one that best suits the ITS goal and the problem involved.

According to AHP, whether an incentive decision is suitable or not to the problem is reflected by the incentive utility with respect to the weight of each incentive element and the historical and real-time sensor readings for each elements from travellers’ previous and current trips. Here, we first apply AHP to weight the incentive element, i.e., criteria, then calculate the incentive utility based on those weights. Using the reasonable weights calculated by AHP, the contributions of each element to the final decision, made by utility comparisons, can be rationally modelled.

To apply AHP to weight the incentive criteria, the process is closely related to travellers’ sensitivity to a criterion. Specifically, if a traveller is highly sensitive to a criterion, e.g., traffic route, then this means that this traveller has a strong preference on his/her habitual choice with respect to this criterion, i.e., a strong preference for his/her habitual route. We measure the sensitivity of a traveller to a criterion by using a commonly-used scale between one and nine, where ‘1’ indicates the lowest sensitivity and ‘9’ is the highest [17,38]. Table 1 gives the sensitivity measurements based on whether any traveller from T is sensitive to each of the criteria or not as listed in Table 2. According to Table 1, the sensitivity of the emphasized criterion, such as “travel modality” for Travellers 1, 3, *T* in Table 2, is rated within the scale between six and nine and the sensitivity of a less-emphasized criterion, such as “travel volume”, for Travellers 1, 3 in Table 2 is rated within the scale between one and five.

Based on the scaled sensitivity of a traveller to a criterion, pairwise comparisons are carried out between all pairs of criteria for each traveller to evaluate the relative importance of one criterion over another to the traveller. For example, considering traveller *t*, the pairwise comparisons result in a square matrix *c* of 3×3, where $c_{ij}$ denotes the pair comparison between criteria *i* and *j* as shown in Equation (2).

(2) $$\begin{array}{c} c=\left(\begin{array}{ccc} c_{1,1} & c_{1,2} & c_{1,3}\\ c_{2,1} & c_{2,2} & c_{2,3}\\ c_{3,1} & c_{3,2} & c_{3,3}\; \end{array}\right)=\begin{array}{c} \left(\begin{array}{ccc} 6/6 & 6/7 & 6/5\\ 7/6 & 7/7 & 7/5\\ 5/6 & 5/7 & 5/5 \end{array}\right) \end{array} \end{array}$$

Based on Equation (2), for the given traveller *t*, the eigenvector for each criterion, say criterion *k*, can be calculated using the geometric mean method as:

(3) $$\begin{array}{c} e_{k}=\sqrt[{3}]{\left(c_{k,1}\times c_{k,2}\times c_{k,3}\right)},\;k=1,2,3; \end{array}$$

According to Equation (3), the normalization of $e_{k}$ will determine $\omega_{t}^{k}$ as the weight of criterion *k* for the traveller *t*, which is formulated as:

(4) $$\begin{array}{c} \omega_{t}^{k}=\frac{e_{k}}{\sum_{k=1}^{3}e_{k}},\;\;k=1,2,3; \end{array}$$

### 3.3. Incentive Utility and Optimization Problem

On the historical and real-time data of travellers and the weights of incentive criteria, we define the utility of a traveller *t* choosing an incentive $I_{ij}$ as:

(5) $$\begin{array}{c} U_{tij}=\omega_{t}^{1}\frac{R_{ti}}{R_{ti}^{max}}+\omega_{t}^{2}\frac{R_{tj}}{R_{tj}^{max}}+\omega_{t}^{3}\left(1-\frac{V_{i}}{V^{max}}\right),\;i\in N,\;j\in M; \end{array}$$

where $R_{ti}$ is the historical times traveller *t* travels through route *i* and $R_{tj}^{max}$ is the maximal times of a traveller in group T travelling through one route in group N. $R_{tj}$ is the historical time of traveller *t* taking modality *j*, and $R_{tj}^{max}$ is the maximal times of a traveller in group T taking one modality in group M. $V_{i}$ is the existing traffic volume of route *i*, and $V^{max}$ is the maximal traffic volume out of all the candidate routes. The utility formulated in Equation (5) denotes that if a traveller receives an incentive suggesting a less congested route or their habitual route or modality, the utility of this incentive to the traveller will be relatively high.

The optimization problem in this paper therefore is to maximize the overall incentive utilities of all the travellers in T, which is formulated as:

(6) $$P:\begin{array}{c} \max\\ \alpha \end{array}\sum_{t=1}^{T}\sum_{i=1}^{N}\sum_{j=1}^{M}\alpha_{tij}\cdot U_{tij}$$

(7) $$s.t.\;\left(\sum_{t=1}^{T}\sum_{j=1}^{M}\alpha_{tij}+V_{i}\right)\leq V_{i}^{max},\forall i\in N$$

where P is subject to the fact that the traffic volume of each route should not exceed its maximal allowed capacity, e.g., $V_{i}^{max}$, as formulated in Equation (7).

## 4. The Decision Tree Method

The ID3 algorithm as the classical decision tree algorithm can help travellers make incentive-based decisions more effectively and solve problem P. ID3 carries out a data mining-based training using objective travellers’ historical sample data to learn a pattern as the output. The pattern could reflect travellers’ common sense behaviours. The learnt pattern is denoted as an incentive decision tree in this paper, which therefore answers the question in which case a traveller tends to be prone to select a specific incentive. With the incentive decision tree, any traveller can easily make his/her incentive decision according to his/her future adopted values of related incentive criteria. The usability of the incentive decision tree is highly related to the design of ID3 and the historical sample data of all the objective travellers used in the training.

### 4.1. Entropy and Information Gain Used in ID3

The key problem here is how ID3 builds the incentive decision tree by learning the pattern incentive issued to a traveller, which is indirectly reflected by the sample data. To solve the problem, ID3 utilizes the entropy of the groups and sub-groups of sample data and the information gains of incentive criteria to decide which criterion to be put at a specific node to work as the decisional criterion to classify sample data in the incentive decision tree. ID3 will successfully build the decision tree after all the branches of each incentive criteria have been considered and the sample data have been classified into a number of relative consistent sub-groups by these incentive criteria.

In order to explain this methodology, we present Table 3 with examples of the sample data of travellers, which have route, modality and route volume as the incentive criteria and travellers’ co-related historical decisions (i.e., to travel or not) as the labels. As an example, in Table 3, we define that the route criterion has three candidate routes, i.e., Routes 1, 2, 3 as the optional values, the modality criterion has three modalities, i.e., car, bus and bike as the optional values, and the route volume criterion has three states, i.e., sparse, medium and congest as the optional values. Accordingly, for the problem formulation given earlier, each criterion in Table 3 could have somewhat more optional values in practice. However, in order to explain how ID3 helps travellers to make an incentive decision in a relatively simple manner, we only need to consider those limited numbers of optional values as shown in Table 3.

In ID3, entropy is used to evaluate the consistency of a group. Considering the sample data group *S* in Table 3, the entropy is formulated as:

(8) $$\begin{array}{c} Entropy\left(S\right)=-p_{⊕}\cdot log_{2}p_{⊕}-p_{⊖}\cdot log_{2}p_{⊖}; \end{array}$$

where $p_{⊕}$ is the ratio of the number of data entries with a positive label, i.e., Yes to travel, for the total number of the data entries in group *S*. $p_{⊖}$ is the ratio of the number of data entries with a negative label, i.e., No to travel, for the total number of the data entries in group *S*. Specifically, as shown in Table 3, there are nine data entries with a positive label and seven data entries with a negative label. Therefore, $p_{⊕}$ to group *S* is 9/16, and $p_{⊖}$ to group *S* is 7/16; and $Entropy\left(S\right)=Entropy\left(\left[9+,7-\right]\right)=-\left(9/16\right)log_{2}\left(9/16\right)-\left(7/16\right)\cdot log_{2}\left(7/16\right)=0.9887$. Generally, the higher the entropy is, the lower the consistency of a sample data group is. For the worst case, where a sample data group having half of the data entries with a positive label and another half of the data entries with a negative label, the group will have the highest entropy reflecting the group having the lowest consistency, i.e., $Entropy\left(S\right)=-\left(1/2\right)log_{2}\left(1/2\right)-\left(1/2\right)\cdot log_{2}\left(1/2\right)=1$.

Based on the entropy of sample data group *S*, we define the information gain of an incentive criterion, e.g., route, modality or route volume in Table 3, to denote how capable this incentive criterion is at classifying group *S*. Generally, if an incentive criterion can classify *S* to have a substantial amount of decrement for substantially decreasing the expected entropy of group *S*, the information gain of the incentive criterion will be high. The information gain of an incentive criterion *A* considering sample data group *S* is formulated as:

(9) $$\begin{array}{c} Gain\left(S,A\right)=Entropy\left(S\right)-\sum_{\upsilon \in Values(A)}\frac{|S_{\upsilon }|}{\left|S\right|}Entropy\left(S_{\upsilon }\right); \end{array}$$

where Values(A) is the group of all the optional values of incentive criterion *A*. $S_{\upsilon }$ is the sub-group of group *S*, in which the data entries all have an incentive criterion *A* for optional value υ, i.e., $S_{\upsilon }=\left\{s\in S|A\left(s\right)=\upsilon \right\}$. In Equation (9), the first part is the entropy of group *S*, while the second part is the sum of the weighted entropies of the sub-groups classified by incentive criterion *A* with known optional values from group Values(A). The weight of each sub-group is the number of the data entries in the sub-group, e.g., $S_{\upsilon }$ for the total number of the data entries in group *S*, e.g., $\frac{|S_{\upsilon }|}{\left|S\right|}$. Therefore, Gain(S,A), as the information gain of incentive criterion *A* for group *S*, is the expected entropy decrement caused by incentive criterion *A* classifying group *S*.

### 4.2. ID3 Working Procedure

**Figure 2:**
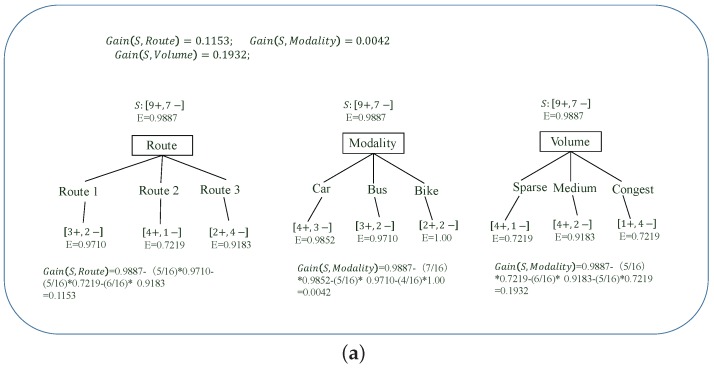

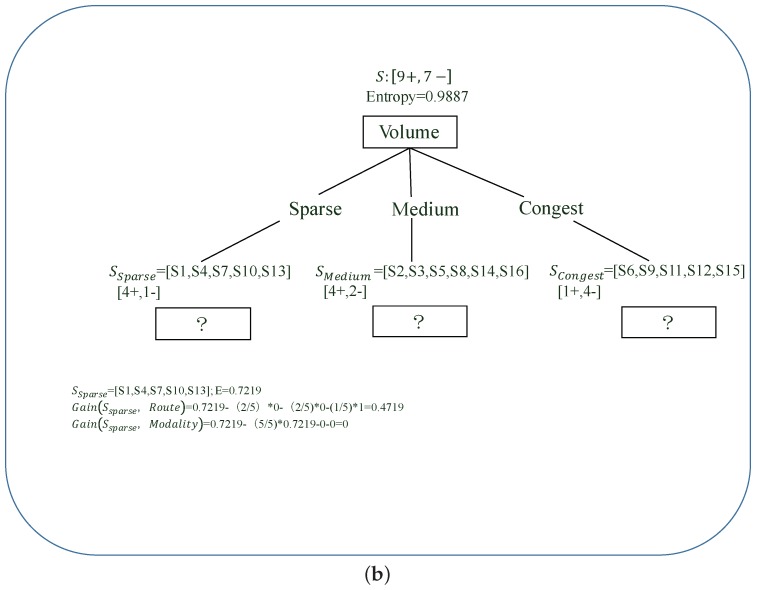
ID3 is used to build the incentive decision tree according to the sample data for group *S* (Step 1).

**Figure 3:**
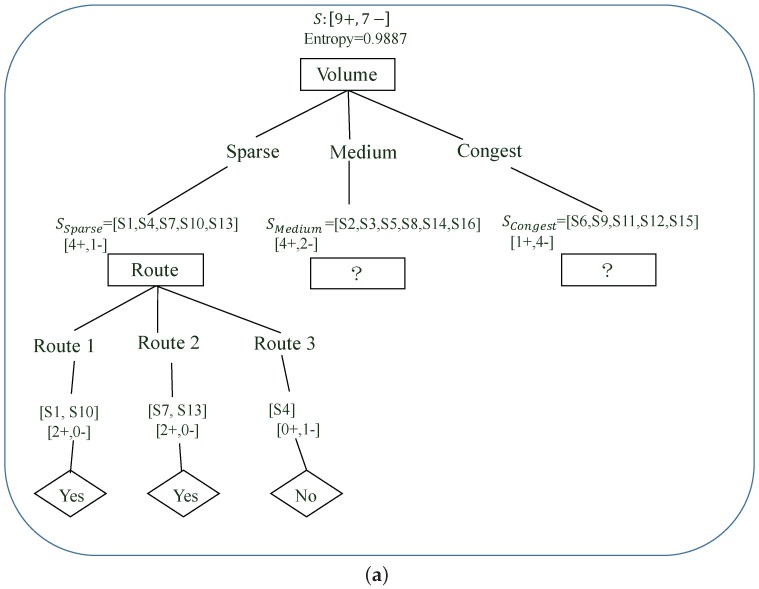

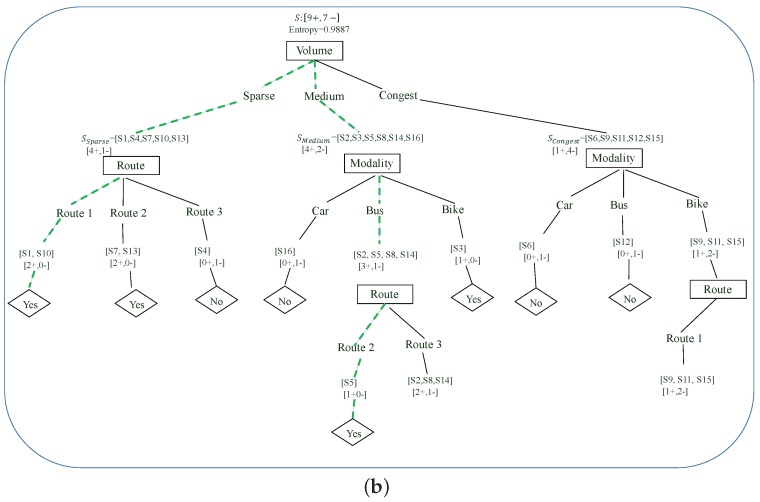
ID3 is used to build the incentive decision tree according to the sample data for group *S* (Step 2).

According to the definition of entropy and information gain, we can carry out ID3 to build the incentive decision tree through a recursive method. Specifically, at the beginning of the ID3 algorithm, the information gain of each incentive criteria to group *S* can be calculated according to Equation (9), which is shown in Figure 2a. Based on this information gain, obviously the route volume incentive criterion should be picked as the best fit decisional incentive criterion to classify group *S* at the first node, as shown in Figure 2b. This is because the route volume incentive criterion has the highest information gain, i.e., 0.1932 compared to the route and modality incentive criterion. For the route volume incentive criterion, group *S* is then classified into three sub-groups, i.e., $S_{Sparse},S_{Medium}$ and $S_{Congest}$, each of which co-relates to one of the optional values of the route volume incentive criterion, i.e., sparse, medium and congest, as shown in Figure 2b. In the next step, ID3 has to decide the next incentive criterion to further classify each of those sub-groups. As shown in Figure 3a, for group $S_{Sparse}$, it has to decide which as yet unprocessed incentive criterion, i.e., modality or route, is to be put forward at the following node to further classify $S_{Sparse}$. In order to do so, ID3 has to recursively carry out the same procedure as for group *S* to pick the un-processed incentive criterion with the highest information gain to be the next decisional incentive criterion of group $S_{Sparse}$. For example, in Figure 3a, the route incentive criterion which has the highest information gain is put at the following node of $S_{Sparse}$. Then, $S_{Sparse}$ is further classified into three sub-groups co-relating to the three optional values of the route incentive criterion, i.e., Routes 1, 2 and 3. The recursive procedure is also carried out for sub-group $S_{Medium}$ and $S_{Congest}$ to further classify their sample data.

ID3 will finish, i.e., converge and get the complete incentive decision tree after all the branches of each incentive criteria have been considered and the sample data have been classified into a number of relative consistent sub-groups by those incentive criteria after a finite number of recursive steps. That means each final sub-group should have a relatively low entropy value. For example, in Figure 3a, after group $S_{Sparse}$ is classified using the route incentive criterion, the obtained sub-groups are all consistent, each of which either has its data entries all having positive labels or negative labels. With the final incentive decision tree, further travellers can make incentive decisions according to their adopted values for their related incentive criterion. For example, according to Figure 3b, if a traveller takes an incentive suggestion for Route 2 with bus as the modality, this incentive will be selected by the traveller if Route 2 has a medium volume. Another example is that if a traveller takes an incentive suggesting Route 1 with any modality, the incentive will be selected by the traveller if Route 1 has a sparse volume. These two examples are demonstrated as the decision making roadmaps shown as the dashed lines in Figure 3b. Hence, this ID3-based incentive decision making can help the RIF solve the incentive problem.

## 5. The PE Method

To solve problem P apart from the decision tree method, we designed another PE-based algorithm for personal incentive generation as Algorithm 1, which is based on a series of comparisons of incentive utilities, instead of data mining on a large amount of historical traffic data. Algorithm 1 carries out the incentive generation for all the travellers in group T. In the beginning of the algorithm, it employs a greedy approach such that each traveller selects an incentive out of all the candidate incentives that have the highest utility (Steps 3–8). The incentive utilities are calculated according to Equation (5). Then, the travellers with the same incentive selected will form an incentive population (Step 6). For example, $C_{ij}$ is the incentive population formed by all of the travellers selecting incentive $I_{ij}$. Afterwards, the incentive utility of each traveller and average utility of each incentive population, e.g., ${\bar{U}}_{ij}$ of $C_{ij}$, will be updated and calculated using Steps 9 and 10. Obviously, the greedy incentive selection will lead to traffic congestion and cause the incentive utilities to drop down.

After the greedy incentive selection, Algorithm 1 runs a finite loop to obtain the optimized incentive results from Step 12 to 23. As shown in Step 12, if an incentive population $C_{ij}$ has homogeneous utility ${\overset{ˇ}{U}}_{ij}$ smaller than its previous average utility, i.e., ${\overset{ˇ}{U}}_{ij}<{\bar{U}}_{ij}$, then this means the population $C_{ij}$ is not in its optimal situation and some travellers in $C_{ij}$ are not satisfied with an incentive $I_{ij}$. Unsatisfied travellers could be those being affected by a congested traffic route *i* or the ones not taking route *i* or modality *j* as their preferred choices. The homogeneous utility of an incentive population ${\overset{ˇ}{U}}_{ij}$ represents the minimal utility, i.e., payoff of the entire incentive population. According to PE, if an incentivized population has a higher homogeneous utility, the incentivized population is more attractive to travellers. On the contrary, in the case of ${\overset{ˇ}{U}}_{ij}<{\bar{U}}_{ij}$, Algorithm 1 has to adjust population $C_{ij}$ and search the travellers in population $C_{ij}$ to find specific travellers to be moved to another population, which will potentially benefit population $C_{ij}$ while not jeopardizing the incentive utilities of the receiving population. The travellers to be moved are the ones that have a higher incentive utility in the receiving population than in the current population. For example, if traveller *t* in incentive population $C_{i^{\prime }j^{\prime }}$ has $U_{tij}<U_{ti^{\prime }j^{\prime }}$ (Step 15), then traveller *t* will be moved to the alternative incentive population: $C_{i^{\prime }j^{\prime }}$ (Steps 16 and 17). After the travellers are relocated, the incentive utilities of all travellers and the homogeneous utilities of all incentive populations will be updated (Steps 20 and 21). Algorithm 1 will finish, i.e., the PE game converges to an equilibrium, when the homogeneous utilities are higher than the average utilities of all the incentive populations or the algorithm runs out of its allowed steps $S^{max}$. Then, $\alpha_{tij}(\forall t\in T,\forall i\in N$, ∀j∈M) as the result of the algorithm is obtained, which allocates incentives to the right travellers.

**Algorithm 1:** The PE-based Algorithm for personal incentive generation.
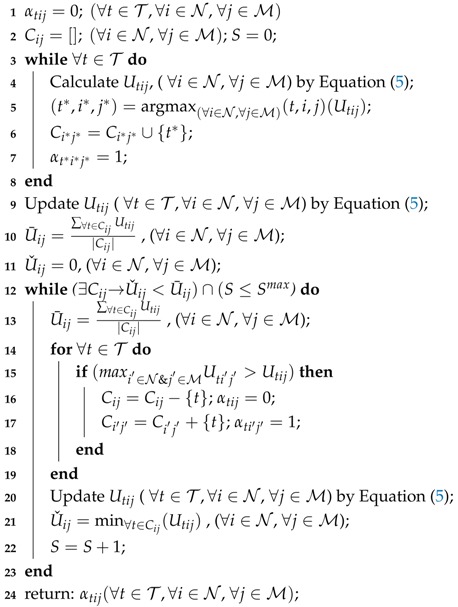


## 6. Analysis of the Decision Tree and PE Methods

This paper provides two different solutions to the personal incentive problem. In this section, we discuss their pros and cons with respect to usability, scalability and computation complexity.

In theory, the decision tree method can effectively provide a solution to the personal incentive problem. In practice, the ID3 algorithm as a classical decision tree algorithm takes travellers’ historical travel data into account to explore travellers’ common sense behaviours. Through ID3, the incentive distribution for each traveller is not based on a heuristic searching algorithm such as the PE method, but is based on data mining of a large amount of travellers’ historical data. As long as the historical data used in the training are at the big data level, the ID3 algorithm will help each traveller to select the best fit incentive, instead of by random attempts. This enables the ID3 algorithm to closely reach the global optimization and not fall into a backwards or forwards dilemma. However, the ID3 algorithm relies heavily on travellers’ historical data and behaviour. According to [26], the ID3 algorithm applied to the incentive problem still faces the problem of overfitting, false data, etc. Considering scalability and computational complexity, the time complexity of ID3 algorithm is O(n), where *n* is the number of sample data used in the training. If the sample data reach a big data level, the algorithm execution time will be enormous and require distributed computing to guarantee finishing the computation in a short time. This will limit the scalability for the use of the ID3 algorithm. This paper only considers a simple scenario with two traffic points, where the ID3 algorithm is affordable for RIF. However, when applying the ID3 algorithm at the smart city level with a large number of pairs of traffic points to be considered, the requirement of heavy computing will limit the application of the decision tree method.

According to Algorithm 1, the main activities of the PE method are to carry out heuristic searching to make sure each incentive population has member travellers with relatively high utilities. The PE method does not depend on a large amount of historical data, but works out the personal incentive problem using an evolutionary game with a series of utility comparisons. It involves less computation and training time compared to the decision tree method, but gives a better result, which is validated by simulations in the next section. However, there are two challenges concerning the PE method. First, there is a possibility that the heuristic search cannot find the optimal solution, but only find a local optimization. This is because the heuristic searching is for each traveller, out of the travellers’ incentive population, without looking at the global problem P. Even through the PE game itself tries to reach a global equilibrium to benefit all of the travellers, this issue still exists. Second, the heuristic search may cause the travellers’ incentive population regrouping to fall into a backwards versus forwards dilemma, where a traveller may be regrouped back to its previous incentive population without reaching an optimization. Those two challenges will escalate if the traveller group T, route group N and modality group M are large, leading to Algorithm 1 not being able to converge after a long time of running. The time complexity of Algorithm 1 is $O(n^{3})$, where *n* is the size of the travellers under consideration. Compared to the decision tree method, the PE method requires much less computation, as the number of travellers between two traffic points is relatively low. This makes the PE method more suitable to work as part of a smart city initiative.

## 7. Simulations

To validate the decision tree and PE methods in RIF, we apply a series of MATLAB-based simulations with the configurations listed in Table 4. Based on the configurations, we run the ID3 algorithm and Algorithm 1 a finite number of times and list the overall incentive utilities of all the travellers as the outputs in Table 5. We implement the greedy method mentioned in Section 5 as a baseline solution to validate our personal incentive solutions. For the greedy method, each traveller will always choose the incentive that suggests his/her habitual route and modality. The greedy method is prone to cause a travel bias without promoting its effectiveness and green transportation use.

According to the outputs in Table 5, it is obvious that the PE method gives the best performance, while the decision tree method gives more of a mediocre performance. For the greedy method, because every traveller takes a selfish approach without cooperation, it causes route congestion and a modality bias that worsens the performance. The PE method outperforms the decision tree method because it utilizes a sophisticated evolutionary game theory and the use of recent historical data of travellers to more effectively, in real time, work out a traveller’s strategy. In contrast, the decision tree method depends heavily on a large amount of historical data and lacks timeliness and cooperation.

**Figure 4:**
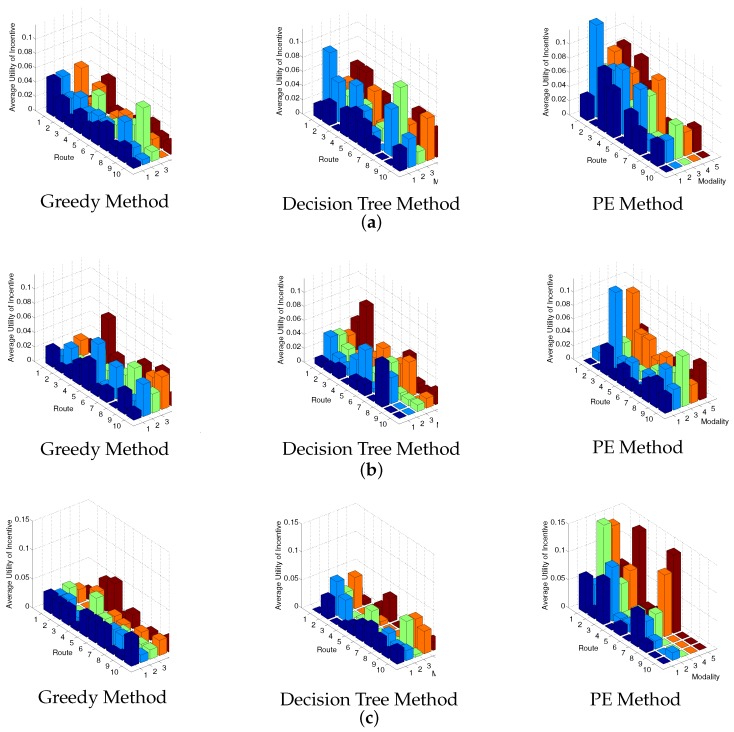
Comparisons of the average utility of incentive using different methods. (**a**) Traveller Scenario 1; (**b**) Traveller Scenario 2; (**c**) Traveller Scenario 3.

To better demonstrate the outputs, Figure 4 demonstrates the average utility of each incentive population provided by different methods, considering different traveller scenarios respectively. Figure 4 demonstrates the same numerical results as summarized in Table 5. According to the outputs, it is clear that the average utility of all the members in an incentive population is improved mostly by the PE method, then medially by the decision tree method, compared to the greedy method.

## 8. Conclusions and Future Work

This paper provides a Rule-based Incentive Framework (RIF) implemented using the decision tree and PE methods. By solving the personal incentive problem, RIF can enable each traveller to pursue more effective and green transportation choices as part of a smart city initiative. The decision tree and PE methods both have their pros and cons. Generally, the PE method gives a better performance through the use of evolutionary game theory and real-time travel information. However, the PE method may not reach the best optimization. The decision tree method depends heavily on the historical data, but it will give effective results if a large amount of sample data is provided.

For future work, we aim to combine the merits of the machine learning methodology, such as decision tree, with game theory, like PE, to come up with more effective intelligent personal incentive solutions, under more complicated system scenarios. In the meantime, we will consider the timeliness of RIF and the acceptance of personalized incentives by travellers, which will make RIF more applicable.

## Figures and Tables

**Table 1:** Travellers’ sensitivity measurements.

Criteria	Travellers’ Sensitivity Measurements						
	Traveller 1	Traveller 2	Traveller 3	...	Traveller *t*	...	Traveller *T*
Travel Route	6	4	9	...	6	...	5
Traffic Volume	1	7	3	...	7	...	8
Travel Modality	9	2	8	...	5	...	7

**Table 2:** Is a traveller sensitive to a criterion?

Criteria	Traveller Sensitive to Each Criterion?						
	Traveller 1	Traveller 2	Traveller 3	...	Traveller *t*	...	Traveller *T*
Travel Route	Yes	No	Yes	...	Yes	...	No
Traffic Volume	No	Yes	No	...	Yes	...	Yes
Travel Modality	Yes	No	Yes	...	No	...	Yes

**Table 3:** The sample data of travellers (grouped as *S*) applied in the ID3 algorithm.

Samples	Incentive Criteria			Travel or Not?
	Traffic Volume	Traffic Modality	Traffic Route	
S1	sparse	car	route 1	Yes
S2	medium	bus	route 3	No
S3	medium	bike	route 2	Yes
S4	sparse	car	route 3	No
S5	medium	bus	route 2	Yes
S6	congest	car	route 3	No
S7	sparse	car	route 2	Yes
S8	medium	bus	route 3	Yes
S9	congest	bike	route 1	No
S10	sparse	car	route 1	Yes
S11	congest	bike	route 1	Yes
S12	congest	bus	route 3	No
S13	sparse	car	route 2	Yes
S14	medium	bus	route 3	Yes
S15	congest	bike	route 1	No
S16	medium	car	route 2	No

**Table 4:** Configurations for the simulation validation.

Parameter	Value
Number of candidate routes: N	10
Number of candidate modalities: M	5
Number of travellers: T in Traveller Scenario 1	100
Number of travellers: T in Traveller Scenario 2	300
Number of travellers: T in Traveller Scenario 3	500
Capacity of each route	75–100
Existing traffic of each route	10–90
Sensitivity measurements for sensitized incentive criteria	6–9
Sensitivity measurements for non-sensitized incentive criteria	1–5
Portion of travellers sensitive to route	50%
Portion of travellers sensitive to modality	50%
Portion of travellers sensitive to traffic volume	100%
$S^{max}$ in Algorithm 1	20
Number of entries of a traveller’s sample data	1000–2000

**Table 5:** Performance comparisons of different personal incentive solutions.

Time of Running	Overall Incentive Utilities of All the Travellers (Traveller Scenarios 1/2/3)		
	Greedy Method	Decision Tree Method	PE Method
1	45.31/129.15/188.38	57.71/150.93/215.35	70.31/170.55/284.88
2	43.93/106.43/208.68	47.00/118.73/216.85	71.13/160.35/290.22
3	49.02/141.33/185.33	57.61/173.27/207.75	74.88/188.97/271.80
4	51.11/134.49/213.11	64.41/164.53/280.10	74.95/176.71/287.78
5	44.77/122.87/177.50	54.93/137.37/187.59	68.18/172.69/273.47
6	46.43/130.33/202.37	55.04/154.25/210.75	72.33/177.83/285.04
7	49.69/138.63/207.96	65.19/147.09/256.82	74.40/189.28/317.79
8	50.11/136.73/203.51	58.29/142.00/284.15	75.94/191.03/290.26
9	42.38/136.70/176.91	51.90/158.81/195.06	64.99/184.28/280.25
10	53.85/135.71/204.07	66.11/140.65/227.70	79.55/184.74/306.97
